# Snake River sockeye and Chinook salmon in a changing climate: Implications for upstream migration survival during recent extreme and future climates

**DOI:** 10.1371/journal.pone.0238886

**Published:** 2020-09-30

**Authors:** Lisa G. Crozier, Jared E. Siegel, Lauren E. Wiesebron, Elene M. Trujillo, Brian J. Burke, Benjamin P. Sandford, Daniel L. Widener

**Affiliations:** Northwest Fisheries Science Center, National Marine Fisheries Service, NOAA, Seattle, Washington, United States of America; University of Nevada, Reno, UNITED STATES

## Abstract

In 2015, the Pacific marine heat wave, low river flows, and record high water temperatures in the Columbia River Basin contributed to a near-complete failure of the adult migration of endangered Snake River sockeye salmon (*Oncorhynchus nerka*, NOAA Fisheries 2016). These extreme weather events may become the new normal due to anthropogenic climate change, with catastrophic consequences for endangered species. Existing anthropogenic pressures may amplify vulnerability to climate change, but these potential synergies have rarely been quantified. We examined factors affecting survival of endangered sockeye (*Oncorhynchus nerka*) and threatened Chinook salmon (*O*. *tshawytscha*) as they migrated upstream through eight dams and reservoirs to spawning areas in the Snake River Basin. Our extensive database included histories of 17,279 individual fish that migrated since 2004. A comparison between conditions in 2015 and daily temperatures and flows in a regulated basin forced by output from global climate models showed that 2015 did have many characteristics of projected future mean conditions. To evaluate potential salmon responses, we modeled migration timing and apparent survival under historical and future climate scenarios (2040s). For Chinook salmon, adult survival from the first dam encountered to spawning grounds dropped by 4-15%, depending on the climate scenario. For sockeye, survival dropped by ~80% from their already low levels. Through sensitivity analyses, we observed that the adult sockeye migration would need to shift more than 2 weeks earlier than predicted to maintain survival rates typical of those seen during 2008-2017. Overall, the greater impacts of climate change on adult sockeye compared with adult Chinook salmon reflected differences in life history and environmental sensitivities, which were compounded for sockeye by larger effect sizes from other anthropogenic factors. Compared with Chinook, sockeye was more negatively affected by a history of juvenile transportation and by similar temperatures and flows. The largest changes in temperature and flow were projected to be upstream from the hydrosystem, where direct mitigation through hydrosystem management is not an option. Unfortunately, Snake River sockeye have likely lost much of their adaptive capacity with the loss of the wild population. Further work exploring habitat restoration or additional mitigation actions is urgently needed.

## Introduction

Climate change is a pervasive threat to biodiversity and ecosystems [1, 2], and the rate of local extinctions [3] and population declines driven by climate change is increasing [4]. Most species do not experience climate stress as their only threat, but in conjunction with numerous other anthropogenic threats [5], which affect their overall vulnerability [6–8]. Recent comparative studies have found that populations experiencing high levels of anthropogenic stress are likely to occur in regions also subjected to especially high exposure to climate change [5, 9]. This convergence of stressors can interact synergistically or antagonistically [10], which makes their combined effects difficult to predict. Thus, despite the clear need to assess co-occurring threats, doing so is challenging because it requires substantial data as well as complex and sophisticated models [11]. Therefore, relatively few projected impacts from climate change quantitatively account for additional stressors.

Widespread declines in European and North American populations of Atlantic salmon have been attributed to climate [12, 13]. Atlantic and Pacific salmon in the contiguous U.S. are similarly at high risk to climate change [8, 14]. Salmon provide a useful case study for exploring relationships among stressors because 1) they are especially vulnerable to climate change through both intrinsic characteristics and pre-existing anthropogenic threats [8] and 2) extensive data exist for quantifying the resulting risk (sensu [15, 16]). Due to the ecological, economic, and cultural importance of salmon, enormous effort has been devoted to tracking these fish individually and mitigating anthropogenic factors that hinder their migration and lifetime fitness. However, more work is needed to synthesize the available data for quantitative examination of how existing anthropogenic threats may compromise resilience to climate change.

Snake River sockeye was listed as an endangered evolutionary significant unit (ESU) in 1991 [17], after having been reduced to a small, mostly resident, residual population [18]. Adults from this ESU, as well as Snake River spring/summer run Chinook salmon (*O*. *tshawytscha*), confront an extraordinarily long and steep adult migration (up to 1450 km with a 2000 m elevation gain). For these species, active migration or multi-month pre-spawn holding periods occur during peak summer temperatures. Thus, the adult spawning migration requires extended exposure to altered climatic conditions. In addition, eight major hydrosystem dams profoundly affect temperatures and flows experienced by salmon in the Columbia Basin [19]. This convergence of pressures may be a harbinger of future biodiversity loss in these unique populations as they respond to climate change. To effectively plan and manage recovery, quantitative projections are urgently needed to assess biological impacts of climate change within the context of efforts to mitigate anthropogenic impacts [20].

In this study, we explored how variation in life history, anthropogenic stressors, and climatic factors combine to affect migration survival of these two ESUs. We modeled the biological impacts of historical and projected temperature and flow on adult migration survival within the larger framework of other anthropogenic impacts. These impacts included hatchery production, transportation of juveniles through the hydrosystem in trucks or barges, fisheries catch, and intricacies of dam passage that generate complex behaviors during the upstream migration.

We also compared the extreme conditions of 2015 to downscaled global climate model (GCM) projections for the 2040s. This comparison provided a check on the extent to which we had to extrapolate beyond the data when projecting future impacts from climate change. It also provided an assessment of the extent to which the consequences observed in 2015 represented a stress test on our preparedness for future conditions. This approach demonstrates the importance of considering co-occurring stressors in modeling biological impacts of climate change. Such consideration will support structured decision making and offer a systematic approach to conserving species within the context of global change [21].

## Methods

Our analysis consisted of five steps. We first conducted retrospective analyses in which we analyzed the apparent survival of fish from each ESU through three consecutive reaches as a function of three covariate types: environmental conditions during the adult migration, adult migration characteristics, and juvenile history. In the second component of retrospective analyses, we modeled arrival timing at the first dam encountered as a function of environmental conditions. Third, we assessed the impact of climate change on adult migration survival utilizing modeled daily temperatures and flows under a historical climate scenario (1929–1998) and two projected future climate scenarios (2030-2059 mean). Fourth, we compared conditions in 2015 with projected mean future conditions. Finally, we conducted extensive sensitivity analyses across a range of assumptions regarding potential changes in arrival timing, temperature, and flow. All analyses were completed in the statistical program R [22].

### Retrospective analyses

#### Tag data

We analyzed detection data from 15,080 Snake River spring/summer Chinook and 2,199 Snake River sockeye salmon marked with passive integrated transponder (PIT) tags. Data were taken from adult migration years 2004-2015 for Chinook and 2008-2017 for sockeye (Table 1, accessed from [23]). All fish had been tagged and released as juveniles, and all had been detected in an adult fishway at Bonneville Dam at least 1 year after tagging. Wild fish constituted 26% of the Chinook dataset and 1% of the sockeye dataset.

**Table 1:** Characteristics of the observation dataset.

MPG	Spring-run Chinook (n)				Summer-run Chinook (n)				Sockeye (n)	Total
	All MPGs	Grande Ronde	Middle Fork Salmon	Upper Salmon	All MPGs	Imnaha	Pahsimeroi	South Fork Salmon	Upper Salmon River	
2004	249	211	15	23	889	242	4	643		**1138**
2005	119	100	7	12	452	133	1	318		**571**
2006	88	75	0	13	309	96	5	208		**397**
2007	98	81	2	15	444	107	3	334		**542**
2008	271	206	2	63	880	222	5	653	14	**1,165**
2009	488	303	44	141	1573	583	73	917	23	**2,084**
2010	751	477	141	133	1337	557	111	669	40	**2,128**
2011	807	533	141	133	1229	543	115	571	516	**2,552**
2012	425	263	66	96	612	245	18	349	122	**1,159**
2013	325	164	63	98	620	160	40	420	205	**1,150**
2014	485	286	58	141	959	296	71	592	343	**1,787**
2015	585	277	56	252	1085	276	58	751	679	**2,349**
2016									183	**183**
2017									74	**74**
**Total**	**4,691**	**2,976**	**595**	**1,120**	**10,389**	**3,460**	**504**	**6,425**	**2,199**	**17,279**
Arrival day		14 May	21 May	22 May		8 Jun	6 Jun	5 Jun	2 Jul	
Hatchery (%)		70	0	52		75	79	86	99	59

Populations within the Snake River spring/summer Chinook ESU are grouped by their median arrival day at Bonneville Dam into spring- and summer-runs. Shown are the median arrival date, total number each year and the percent of hatchery fish from each population.

#### Population groups

While the Snake River sockeye salmon ESU consists of only a single population, the Snake River spring/summer Chinook salmon ESU consists of multiple populations, each with distinct run timing [24]. For salmon, the term “population” often refers to fish from individual streams. Within an ESU, however, populations in nearby streams tend to behave similarly due to shared evolutionary histories and ecological conditions. This hierarchical population structure led NOAA to define major population groups (MPG) for recovery planning, using genetic, ecological, and habitat data for all ESUs listed under the Endangered Species Act [25, 26]. In our analysis, most populations within each major tributary to the Salmon River had similar run-timing, which coincides with MPG designations; therefore, we grouped these populations by MPG for analysis. However, two populations migrated at times distinct from the remainder of their respective MPGs (Imnaha River and Pahsimeroi River), and were thus treated as separate “populations.” We assigned each fish to a population based on juvenile release location. To ensure correct assignment we included only fish that had been released within the boundaries of a single MPG.

**Fig 1:**
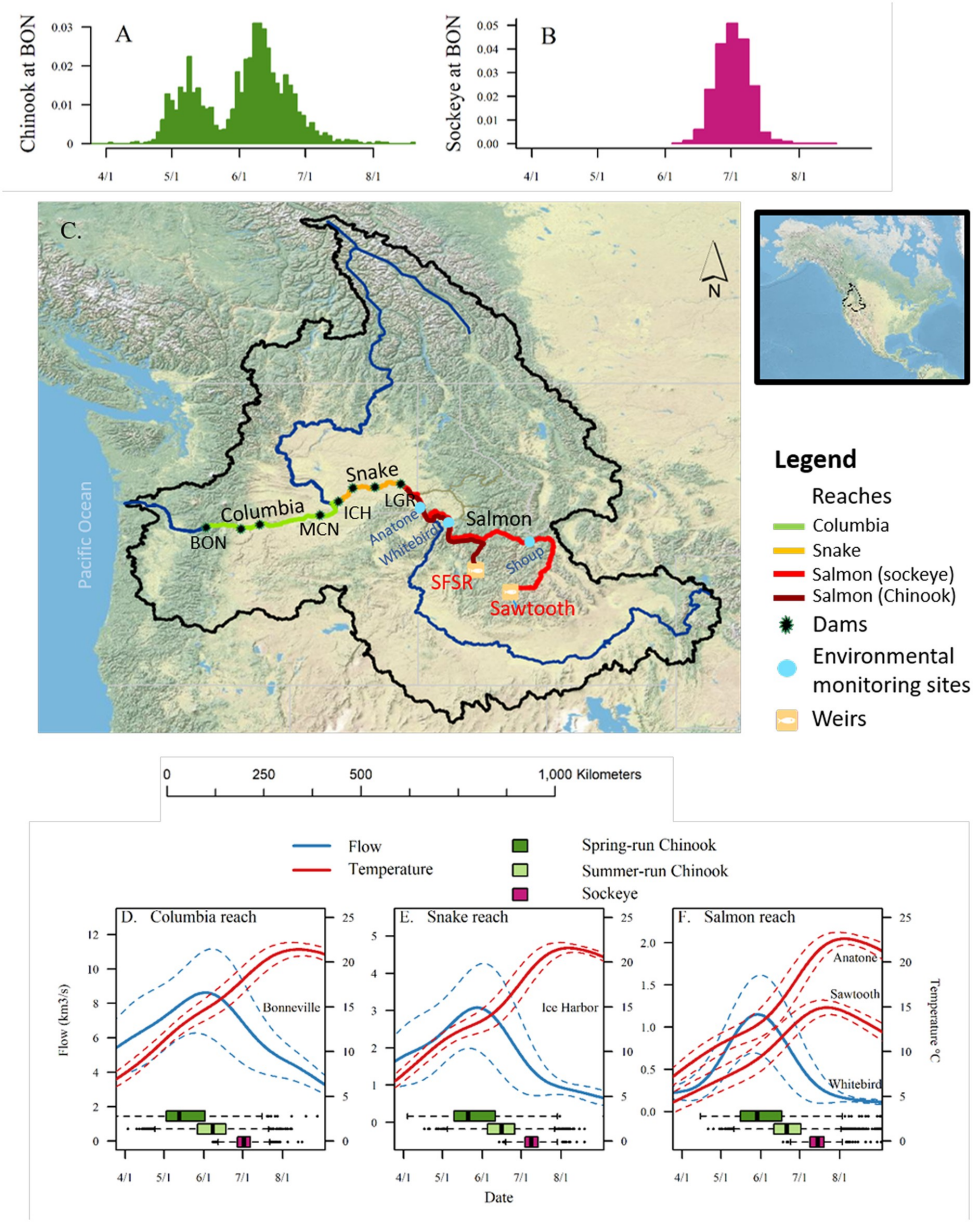
Overview of migration route, timing, and environmental conditions during each run. Histograms showing the frequency distribution of arrival timing of spring/summer Chinook (A) and sockeye salmon (B) at Bonneville Dam (BON). Map (C) shows migration reaches modeled, major dams, and other sites where environmental data were collected. Panels D-F show smoothed mean reach-specific daily mean temperatures and flows (±SD) during 2004-2016 with arrival dates at the respective dams (boxplots) for tagged Snake River spring/summer Chinook and sockeye salmon. For the Columbia reach model (D), we relied on environmental data from Bonneville Dam. For the Snake reach model (E), we utilized environmental data from Ice Harbor Dam. The Salmon reach model (F) used temperature data from Anatone, Washington, and flow data from Whitebird, Idaho. Temperatures at Sawtooth Hatchery are shown for comparison with Anatone to represent the lower temperatures that fish experience at the upper end of the Salmon reach.

MPGs were further grouped into “spring-run” and “summer-run,” based on their arrival timing at Bonneville Dam. Spring/summer run Chinook demonstrate bimodal migration timing (Fig 1A) which results from a characteristic rank order of migration timing by different populations [24]. For populations designated as “spring-run,” arrival at Bonneville Dam peaks in mid-May, whereas arrival of those designated as “summer-run” peaks in June (Table 1, and [27]).

#### Survival models

We modeled apparent survival of Chinook and sockeye salmon through the three separate river reaches: the Columbia reach, from Bonneville to Ice Harbor Dam; the Snake reach, from Ice Harbor to Lower Granite Dam; and the Salmon reach (Fig 1). The estimate of survival included any detections upstream of the reach, and given the high detection efficiency at these dams based on mark-recapture modeling [27, 28] and weirs, was likely close to actual survival.

The Salmon reach extended from Lower Granite Dam to the Sawtooth Valley for sockeye and from Lower Granite to the South Fork Salmon River for Chinook. Although spring/summer-run Chinook spawn in multiple tributaries, we limited our analysis of the Salmon reach to Chinook tagged at sites with high adult detection rates (Johnson Creek and Knox Bridge, within the South Fork Salmon River). Other Chinook spawning tributaries had low detection rates, making survival rates difficult to separate from detection probabilities.

A fish was determined to have entered the reach on the day it was first detected at the dam of reach entry, and to have survived the reach if it was detected again at the upper end of the reach or at any site further upstream. For Chinook and sockeye respectively, detection probability estimates were 99.7 and 98.4% at Lower Granite and 98.9 and 94% at Ice Harbor Dam [27, 28]. Most adult fish with missed detections at Ice Harbor were later detected upstream, because survival between the four dams in the lower Snake River is generally quite high (Table 3 in [28]). At the Sawtooth Fish Hatchery and South Fork Salmon weir, detection probabilities are also believed to be near 100%, or at least unbiased with respect to our covariates.

To inform our models of apparent survival through the hydrosystem and to spawning grounds, we considered a wide range of variables that accounted for climate and anthropogenic impacts. These included environmental variables describing the river environment, variables describing adult migrant characteristics, and variables accounting for juvenile migration history and origin (Table 2).

**Table 2:** Variables used in upstream migration survival models.

Variables affecting adult migration for spring/summer Chinook and sockeye salmon		
Name	Symbol	Description
**Environmental factors**		
Temperature	*T*	Daily mean river temperature on date of detection at reach entry. For Salmon reach, we used mean weekly temperatures at Anatone, WA, starting on arrival day at Lower Granite and extending 1 week for Chinook and 2 weeks for sockeye.
Flow	*F*	Daily mean flow starting on day of detection at reach entry. For Salmon reach, we used flows at Whitebird, ID averaged over 1 week for Chinook and 2 weeks for sockeye.
Cumulative temperature	*M*	Number of degree days (mean temp × duration of exposure) experienced by individual fish prior to entering study reach (i.e., from Bonneville Dam to reach entry).
**Adult characteristics**		
Age	*A*	Number of years a fish spent in the ocean before maturing.
Fishery catch	*C*	Number of fish caught in Zone 6 fishery (lower Columbia R) during week of detection at Bonneville.
Year	*y*	Year of adult migration (random effect).
**Juvenile characteristics**		
Population	*P*	Chinook MPGs: Grande Ronde, Middle Fork Salmon, Upper Salmon, and S Fork Salmon. Imnaha and Pahsimeroi populations analyzed separately based on run timing.
Juvenile migration history	*J*	Whether fish was transported or migrated in-river as juvenile.
Hatchery/ wild	*H*	Hatchery or wild origin.

#### Environmental data

Environmental data for the hydrosystem were collected by the U.S. Army Corps of Engineers at continuous water-quality monitoring stations maintained at or near dams [29]. Upstream from Lower Granite Dam, we used environmental data collected at gage stations operated by the U.S. Geological Survey [30]. We interpolated or regressed missing data from a neighboring dam following the methods described in Crozier, Weisebron [28].

We divided the migration into three reaches for analysis of survival; the Columbia and Snake reaches within the hydrosystem and the free-flowing Salmon reach (Fig 1). Reaches were determined by a combination of detection ability (which is highest at dams) and relatively consistent environmental conditions. Thus, environmental conditions in the Columbia and Snake reaches were well-represented using the conditions upon entry of each reach. For the Columbia and Snake hydrosystem reaches, we assigned values of ***flow*** (*F*) and ***temperature*** (*T*) to individual fish according to their arrival date at Bonneville Dam and Ice Harbor Dam, respectively (Table 2).

Conditions at the entry of the Salmon reach (Lower Granite Dam), on the other hand, were not consistently correlated with environmental conditions throughout the reach. Temperatures at Lower Granite are frequently reduced using releases of cool water from a reservoir on the Clearwater River. Furthermore, flow on the lower Snake River is affected by regulation in the Hells Canyon complex, which does not represent the majority of the migration through the free-flowing Salmon River. To more accurately reflect conditions in the Salmon reach, we used temperature data from a gage located upstream from the Snake River confluence with the Clearwater (Anatone, gage 13334300), and flow data from a gage on the Salmon River at Whitebird, ID (gage 13317000). The temperature record at Whitebird had long stretches of missing data, but the available data were strongly correlated with temperatures at Anatone (*r* = 0.96).

Travel times though the Salmon reach were substantially longer than those in the Snake and Columbia reaches, and depended on the distances from reach entry to spawning tributaries. In our study fish, median travel time through the Salmon reach was 19 d (SD = 13.5) for South Fork Salmon summer Chinook and 39 d (SD = 12.6) for sockeye. To account for the relevant period of exposure to high temperatures for each species, despite not having individual travel time data, we averaged over different temporal extents in our temperature index. South Fork Salmon River Chinook salmon spend about one week within warmest stretch of the Salmon River before entering a spawning tributary [31]. Therefore, for Chinook we used average flow and temperature over the one-week period following detection at Lower Granite Dam. For sockeye, we used average flow and temperature over the 2-week period following the date of Salmon reach entry, which represents the average travel time for sockeye from Lower Granite Dam to the USGS gage at Shoup, Idaho [32]. Temperatures decline as they move upstream, with much cooler temperatures at the Sawtooth weir, shown in Fig 1.

#### Cumulative migration temperature

We included cumulative migration temperature (*M*_*i*_), or degree days accumulated by an individual fish *i* prior to entering a reach, as a separate covariate in the Snake and Salmon reach models (*M*_*Snake*,*i*_, *M*_*Salmon*,*i*_). Because of different thermal conditions in the Columbia and Snake Rivers, we separated these legs as follows:

$$M_{Snake,i}=D_{1,i}*\;\frac{T_{BON,i}\;+\;T_{MCN,i}}{2}\;+\;D_{2,i}*\;\frac{T_{MCN,i}\;+\;T_{ICN,i}}{2}$$ (1)

where *D* is the number of days traversing from Bonneville to McNary (*D*_*1*_) and McNary to Ice Harbor (*D*_2_), and *T* is daily mean temperature on the date of first detection at each dam. Similarly, we calculated the total thermal load from Bonneville to Lower Granite as a predictor of survival through the Salmon reach as:

$$M_{Salmon,i}=M_{Snake,i}+\;D_{3,i}*\;\frac{T_{ICH,i}\;+\;T_{LGR,i}}{2}$$ (2)

where *D*_*3*_ is the number of days traversing from Ice Harbor to Lower Granite, and *T* is the daily mean temperature on the date of first detection at each dam. While most fish migrated quickly through these reaches, slower-moving fish demonstrated a wide range of migration times, creating highly skewed distributions. To limit the leverage of especially slow fish, we log-transformed this variable for modeling.

#### Adult migration characteristics

We considered the ***ocean age*** of fish (*A*), defined as adult detection year minus juvenile migration year, ***fisheries catch*** (*C*), defined as harvest in the Columbia River reach (Zone [6, 33]), and a random effect for year (*y*) as covariates in the survival models. Most of the fish in our database had spent two years in the ocean (66% in Chinook and 89% in sockeye). We considered fish that entered the fishing zone immediately prior to a gillnet opening more likely to be caught in the fishery than fish that migrated during low-catch periods. To account for this differing vulnerability to the fishery, we added an index of catch for the period immediately after the fish passed Bonneville Dam as a covariate in the model. This index came from all sources of reported fishery catch in this reach (assembled by Stuart Ellis, CRITFC and Jeromy Jording, NOAA). We disaggregated catch data into weekly estimates either directly from the data, as for gillnet openings, or by linear interpolation over longer reporting periods, as from hook and line fisheries [27, 28]. Catch (fish harvested per week) was cube-root transformed for modeling to limit the leverage of a small number of high values and render the distribution approximately normal for simulations.

#### Juvenile history

Juvenile covariates were all categorical variables: population, hatchery origin, and juvenile migration history. For ***population*** (*P*), we defined spring-run Chinook as those from the Grande Ronde, Middle Fork Salmon, and Upper Salmon River MPGs. Summer-run Chinook were defined for (*P*) as those from the South Fork Salmon MPG, Imnaha River, and Pahsimeroi River (Table 1). ***Hatchery vs wild origin*** (*H*) was based on designation in PTAGIS [23]. For sockeye, nearly all designations were hatchery origin, and all from a single population; therefore, *H* and *P* were not considered for the Snake River sockeye ESU.

In an attempt to increase juvenile migration survival, the U.S. Army Corps of Engineers transports fish downstream from Lower Granite, Little Goose, or Lower Monumental on the Snake River to below the last dam, Bonneville, on the Columbia River [34]. Thus, we defined ***juvenile migration history*** (*J*) as “transported” for fish whose last juvenile PIT tag detection site was at the entrance to a transport holding raceway. In the absence of detection on a transport raceway entrance, migration history was defined as “in-river.” In our database, 33% of Chinook and 38% of sockeye were transported, and the remaining were assumed to have travelled in the river.

#### Model fitting

We modeled each species/reach combination separately utilizing general additive mixed models (GAMM, [35]) with the MGCV package [36]. The GAMM allows for complex relationships between covariates and for additional unexplained variation to be included as random effects. We parameterized all continuous variables (*T*, *F*, C, and *M*) with smoothers (*s*) fit with thin plate regression splines. To avoid overfitting, we limited knots to 4 for *T* and *F* variables and to 3 for *C* and *M* due to presumed unidirectional relationships (increased catch and higher cumulative temperature exposure are hypothesized to decrease survival). The remaining variables (*P*, *A*, *H*, *J*) were included as categorical factors with *y* as a random effect for each year. Every fish had an individual value for each variable.

We fit a global model for each species/reach utilizing a binomial distribution including all relevant variables where *S*_*i*_ is the survival state of fish (alive or dead) through the modeled river reach:

$$S_{i}\sim s(T_{i})+s(F_{i})+s(C_{i})\;or\;s(M_{i})+P_{i}+A_{i}+J_{i}+H_{i}+y.$$ (3)

Catch (*C*) was considered only in the Columbia reach while *M* was considered only in the Snake and Salmon reach models. We applied GAMM smoothers (*s*) to four covariates, e.g., *s*(*T*_*i*_). We only combined covariates that had a pairwise correlation coefficient less than 0.7 to prevent problems with collinearity. Additionally, we did not consider *P* and *H* in any models for the sockeye ESU because it consisted of only one population dominated by hatchery fish.

Following the fitting of global models, we compared all combinations of variables by Akaike information criterion (AICc) [37] utilizing the dredge function in the MuMIn package [38]. We selected the most parsimonious model based on AICc for further analysis. We also used the area under the receiver operating characteristic curve (AUC) to characterize the goodness of fit of our survival models. In this case, the AUC represents the probability that a randomly chosen positive case (fish that survived) had a higher predicted probability of surviving than a fish that died (negative case).

#### Arrival-time models

To initiate the simulation model, we first needed a model of ***arrival day*** at Bonneville Dam that accounted for well-documented plasticity in this behavior [24, 39]. To achieve this, we fit a retrospective model of arrival timing for both species. Arrival day of Snake River sockeye was approximately normally distributed. We therefore modeled arrival day at Bonneville Dam for sockeye as a single normal distribution where the mean was a function of annual metrics of environmental conditions.

For Chinook, we modelled arrival day as a mixture of two normal distributions, where the mean of each mode (spring or summer) was determined independently by an annual environmental metric. The resulting aggregate distribution of arrival day for Chinook was bimodal, with peaks in mid-May and late-May/early June, as observed. We used population-specific median arrival days to parse populations into *spring* and *summer* groups corresponding to the two modes (for additional analysis, see [27], and Table 1, Fig 1).

The probability density function of arrival day (Pr(x)) at Bonneville dam for Chinook [40] was the weighted average *w*_*k*_ of the predicted distribution for each component of the run *k*:

$$\text{Pr}(x|\theta )=\sum_{k=1}^{2}w_{k}g(x|\theta_{k})$$ (4)

where *g* is a Gaussian distribution, and θ is the vector of parameters for each run. The weights *w*_*k*_ reflected the proportion of spring run (p) and the proportion of summer run (1-p). The parameters θ consisted of the mean and standard deviation of each distribution. The standard deviations were separate for each run, but constant over time. The mean for each distribution (*μ*_*k*,*y*_) varied by year (t):

$$\mu_{\text{k},\text{t}}=\beta 0_{\text{k}}+\beta 1_{\text{k}}{\text{E}}_{\text{k},\text{t}}+\varepsilon ,$$ (5)

where *ε* is the residual error. The regression parameters *β0*_*k*_ and *β1*_*k*_ were fit by maximum likelihood for all possible combinations of each considered covariate (*E*_*k*_), and the resulting model fits were compared by (AICc). The possible combinations consisted of a single covariate for spring-run and a single covariate for summer-run. We compared model fit across all monthly and bimonthly means of temperature or flow at Bonneville Dam during March-June as potential covariates (*E*_*k*_). We selected the model with lowest AICc for further analyses.

### Prospective analyses

#### Temperature and flow projections

Climate scenarios for our prospective analyses were based on work conducted by the federal agencies that manage the Federal Columbia River Power System. The River Management Joint Operating Committee [41] modeled managed flows under three scenarios. The first was a ***historical*** reference period, using observed meteorological conditions from 1929 to 1998. The second and third were projections of climate change for the 2040s, using mean meteorological conditions forecast from 2030 to 2059 under two different GCM scenarios.

The historical simulation differed from observed temperatures and flows during 1929–1998 because all years were modeled by the Bonneville Power Administration’s model, *HYDSIM*, using the modern hydrosystem configuration and operating rules. Naturalized flows that provided input for *HYDSIM* came from the variable infiltration capacity (VIC) hydrological model produced as part of the 2860 Hydroclimate Scenarios Project (available at https://cig.uw.edu/news-and-events/datasets/pnw-hydroclimate-scenarios-project-2860). Climate change projections stemmed from the Coupled Modelling Intercomparison Project 3 [42], which were statistically downscaled using the hybrid delta method [43]. Stream temperatures were then modeled for the regulated-flow scenarios using a state space framework and a semi-Lagrangian numerical scheme using methods described in Yearsley [44] and [45].

The joint committee selected outputs from two GCMs [41] for modeling regulated flows: a high temperature/high flow scenario (MIROC 3.2 A1b) and a moderate temperature/low flow (ECHO G B1) scenario, henceforth referred to as ***wet*** and ***dry***, respectively. Each was then compared to the historical scenario. These wet and dry scenarios were selected to represent the extremes of projected low and high mean annual precipitation change across the entire Columbia River Basin. They were selected from a comparison of 10 GCMs across two emission scenarios (Table 3 in [41]). New projections of naturalized flows from a broader array of recent GCM projections were recently published [46]. These projections compared additional downscaling methods and four hydrological models. The ensemble mean projection across 10 GCMs from the updated carbon emissions scenario (representative concentration pathway 8.5) was very similar to our *wet* scenario when comparing projected mean percent change per day. This comparison suggested that the older scenario was still representative of current climate projections. We chose the older scenarios because they include the full modeling chain including regulated flows and daily stream temperatures needed for biological impact studies, whereas the new scenarios only include naturalized flows at this time.

#### Survival projections

The survival projections involved four steps. First, we used annual metrics from the temperature and flow projections to simulate arrival timing using the selected models. Second, we used the daily time series of temperatures and flows to move fish through the migration using an existing travel time model [47]. The travel time model used mixture models to account for bimodal migration movements at each dam (fast- and slow-moving migrants) as well as the effects of hourly river conditions on migration behavior. The travel time model predicts arrival time at every dam, and hence can be used to align reach-specific environmental variables to each simulated fish.

In the third step, we used fish-specific environmental conditions in each reach to predict survival using the GAMMs with the random effect for year set to zero. Catch, juvenile transportation rate, and the proportion of hatchery fish were simulated to reflect the interannual variability present in the observation dataset. The same survival models were applied to all Chinook, but the different starting distributions produced different survival rates for each run. We reported separate estimates for each distribution and the aggregate run.

Fourth, we simulated the 70-year daily time series for the three climate scenarios (historical, wet, dry) over 200 loops using different parameter estimates for each loop. To account for uncertainty in model fits we drew parameter values for arrival timing and survival models using the *mvnorm* function in the R package *MASS* [48], based on the covariance matrix from the retrospective model fits. We simulated 100 fish per year/scenario/loop/species for a total of 8.4 million modeled fish. The variation in results across simulations therefore characterizes the cumulative uncertainty in all submodels (arrival timing, travel times, and survival models).

### Sensitivity analyses

#### Arrival day

Fish may be able to mitigate the impacts of climate change more than our simulations suggest if they can initiate migration earlier in spring, when temperatures are cooler. To explore this potential, we tested the sensitivity of our simulation to day of arrival at Bonneville Dam. This was done by adjusting the arrival times predicted by our models to 3, 6, 9, 12, and 14 d earlier. Once new arrival dates at Bonneville Dam were determined, migration and survival were re-simulated using the same methods described above.

#### Temperature

Acknowledging that climate projections are updated regularly, we also explored the sensitivity of our results across ranges of environmental change. Sensitivity to changes in temperature was tested by adding a constant delta temperature to the daily time series from existing scenarios and then re-predicting survival in each reach with the new temperature values. Negative delta temperatures implied less warming, whereas positive delta temperatures implied more than predicted by the two selected GCMs. We tested delta values -0.8, -0.4, 0.4, 0.8, 1.2, 1.6, and 2°C.

#### Flow

To test the sensitivity of the simulation to the magnitude of projected changes in flow, we applied a multiplier to the changes in mean daily flow from the dry and wet scenarios. We used proportional rather than absolute change because flow cannot be negative. We used the equation:

$$F_{t,s,x}=F_{t,hist}+\Delta F_{t,s}*F_{x},$$ (6)

where *F*_*t*,*his*t_ is flow in the historical scenario on day *t*, Δ*F*_*t*,*s*_ is the difference in flow between the historical scenario and climate scenario *s* (either dry or wet) on day *t*, and *F*_*x*_ is the sensitivity multiplier, or flow factor. A flow factor of 0 would simply be equal to the historical scenario, while a factor of 1 would equal the previously estimated flow value of that scenario. A factor greater than 1 would represent more extreme change, while a factor less than 1 would represent less extreme change, than in the original scenario. We tested multipliers of 0, 0.2, 0.4, 0.6, 0.8, 1.2, and 1.4. If the multiplier caused flows to be less than 10 m^3^/s, we used 10 m^3^/s as a minimum flow.

## Results

### Retrospective analyses

#### Survival models

Chinook salmon had higher survival overall than sockeye, with mean observed survival across all reaches in the 80–97% range compared with 55–83% for sockeye (S1 Table). Survival through the hydrosystem was lowest in the anomalously warm year of 2015 for both Chinook and sockeye, at 93% of average for spring Chinook, 70% for summer Chinook and 8% for sockeye (S1 Table). For all reach/species combinations, the top model of survival included effects of temperature, and flow was included in nearly all models (Table 3). Goodness-of-fit tests assessed using AUC showed strong performance of the top models, ranging from 0.68 for Chinook in the Salmon reach, to 0.87 for Sockeye in the Snake reach.

**Table 3:** Survival model selection results.

Reach	Equation	df	ΔAICc
Chinook salmon			
Columbia	***s***(***T***) ***+ s***(***F***) ***+ s***(***C***) ***+ H + J + A + y***	**20**	**0.0**
AUC = 0.70	*s*(*T*) *+ s*(*F*) *+ s*(*C*) *+ H + J + A + P + y* (*global*)	25	8.1
	*null*	1	1137.7
Snake	***s***(***T***) ***+ s***(***F***) ***+ s***(***M***) ***+ H + A + y***	**20**	**0.0**
AUC = 0.77	*s*(*T*) *+ s*(*F*) *+ s*(*M*) *+ H + J + A + y*	21	0.1
	*s*(*T*) *+ s*(*F*) *+ s*(*M*) *+ J + A + y*	20	1.6
	*s*(*T*) *+ s*(*F*) *+ s*(*M*) *+ J + A + H + P + y* (*global*)	26	4.0
	*null*	1	552.5
Salmon	***s*(*T*) *+ s*(*F*) *+ s*(*M*) *+ A+ H + y***	**9**	**0.0**
AUC = 0.68	*s*(*T*) *+ s*(*F*) *+ s*(*M*) *+ A + y*	6	0.5
	*s*(*T*) *+ s*(*F*) *+ s*(*M*) *+ J + A + H + P + y* (*global*)	25	5.4
	*null*	1	69.8
Sockeye salmon			
Columbia	***s***(***T***) ***+ s***(***C***) ***+ A + J + y***	**12**	**0.0**
AUC = 0.81	*s*(*T*) *+ S*(*F*) *+ s*(*C*) *+ A + J + y* (*global*)	13	0.9
	*null*	1	861.8
Snake	***s***(***T***) ***+ s***(***F***) ***+ s***(***M***) ***+ y***	**9**	**0.0**
AUC = 0.87	*s*(*T*) *+ s*(*F*) *+ s*(*M*) *+ A + y*	11	1.2
	*s(T*) *+ s(F*) *+ s(M*) *+ J + y*	10	1.8
	*s(T*) *+ s(F*) *+ s(M*) *+ A + J + y (global*)	12	2.7
	*null*	1	228.7
Salmon	***s(T***) ***+ s(F***) ***+ s(M***) ***+ y***	**10**	**0.0**
AUC = 0.73	*s(T*) *+ s(F*) *+ s(M*) *+ A + y*	11	0.1
	*s(T*) *+ s(F*) *+ s(M*) *+ J + y*	11	1.3
	*s(T*) *+ s(F*) *+ s(M*) *+ J + A + y (global*)	12	1.6
	*null*	1	129.3

All combinations of variables in the global models were compared, and are shown with the null model and all models with ΔAICc < 2. We selected the simplest model with ΔAICc < 2, shown in bold. The area under the curve (AUC) is also shown for each model.

In the Snake and Salmon reaches, the top model for both species accounted for the temperature load that accumulated prior to reach entry. In addition to environmental effects, fish age, catch, and juvenile transportation had negative effects on survival for both species. The survival rate for hatchery Chinook was lower than for wild Chinook in all three reaches, although the effect size was small in the Snake and Salmon reaches.

**Fig 2:**
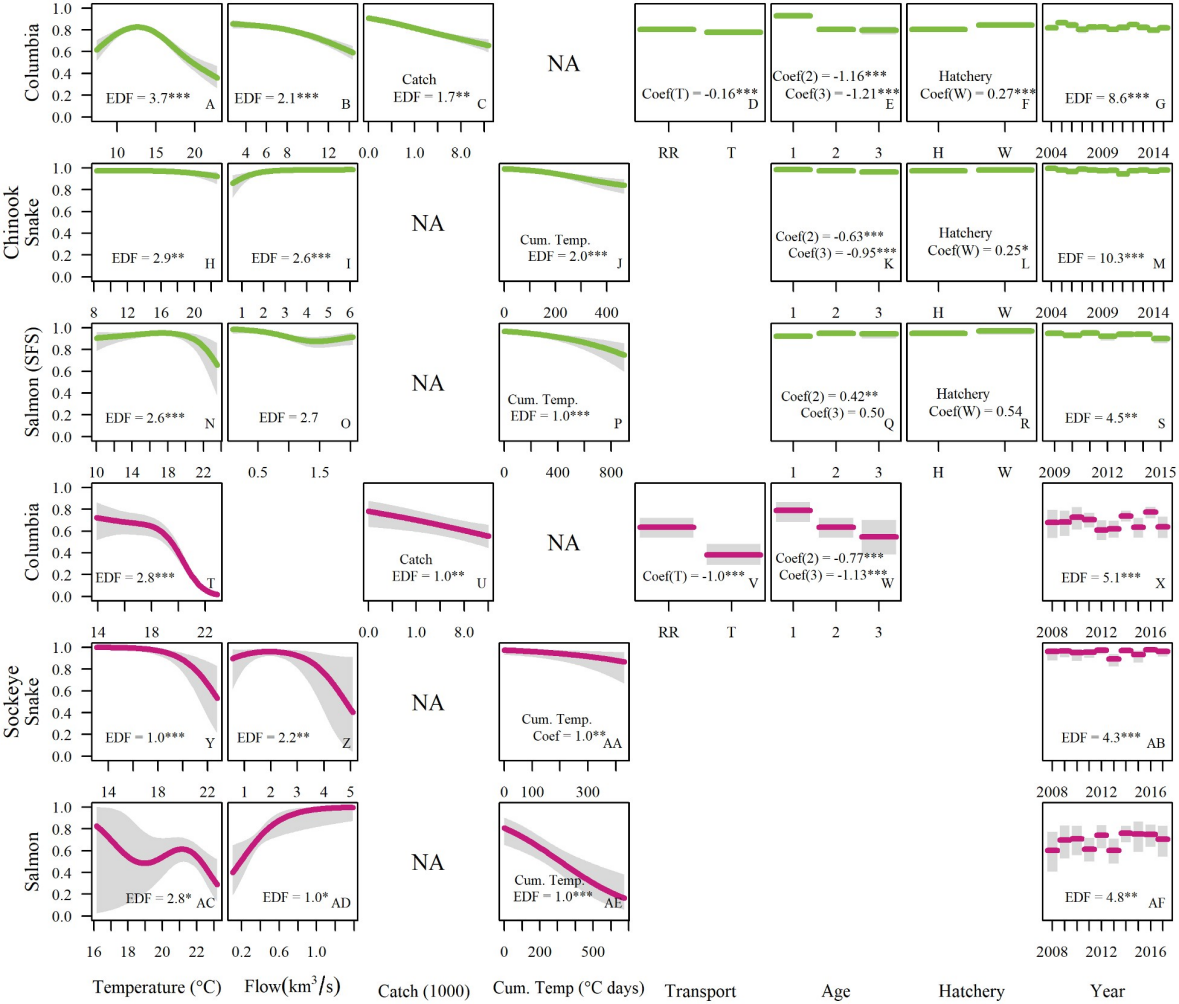
Main effects on predicted survival probabilities of the top migration survival model for each reach. The modeled effects of each variable are shown while other variables are set at their mean (continuous variables) or most common (factor variables) value. Estimated degrees of freedom (EDF) or coefficient values (Coef.) are shown for each variable (significance levels: * = P < 0.05, ** = P < 0.01, and *** = P < 0.005).

**Fig 3:**
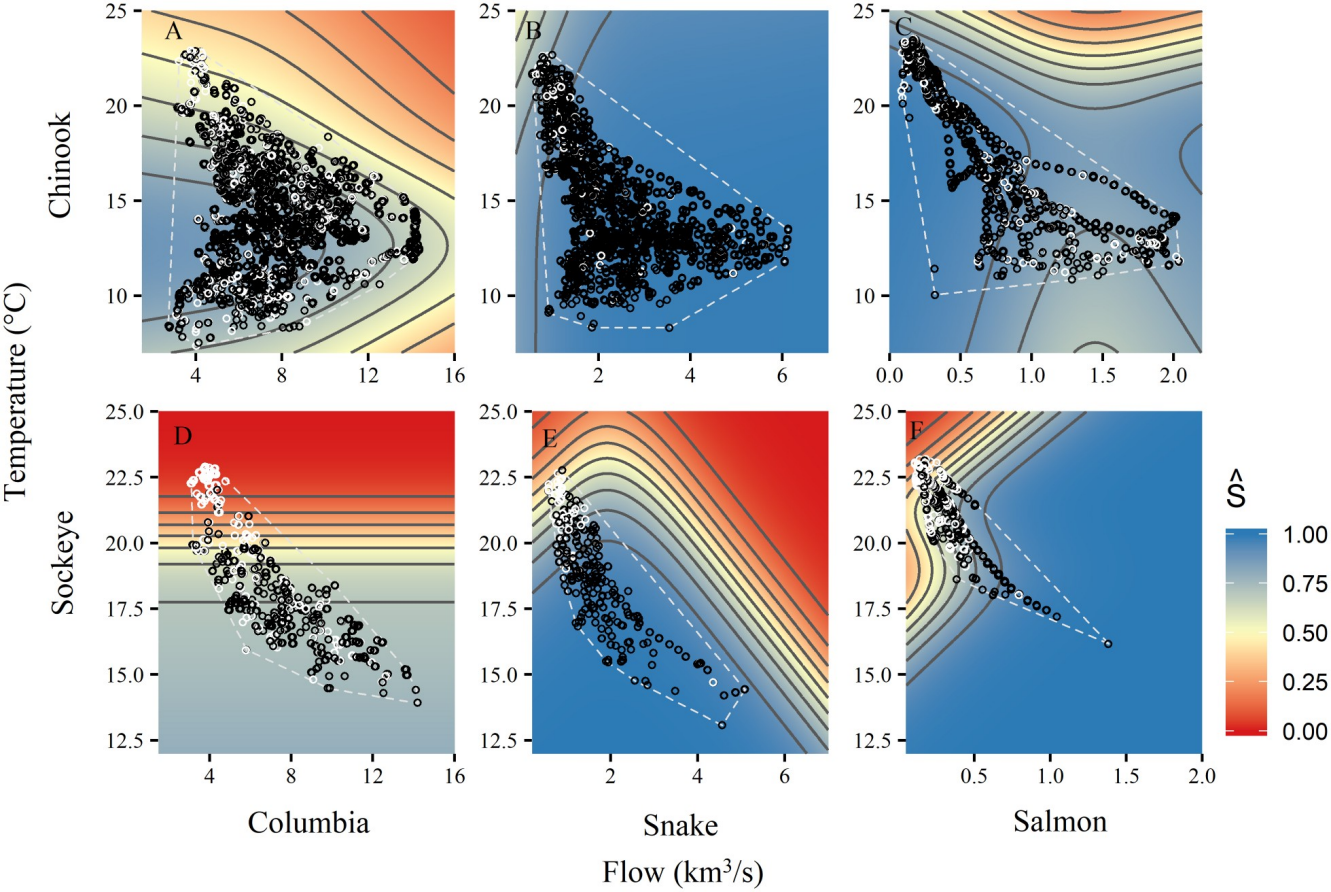
Conditional surfaces of model-predicted survival ($\hat{\;S}$) with variation in temperature and flow. Contour lines show incremental changes in predicted survival of 10%. The white polygons characterize the range of conditions fish experienced in the observation dataset for comparison to simulated conditions. All other variables were set at median values (or most common value for factors) to produce surfaces. Points indicate the combinations of temperature and flow at which individual fish were detected at reach entry and were either detected (black) or not detected (white) upstream.

Survival of both species declined as temperature increased above 15°C (Figs 2 & 3). The temperature effect was not always unidirectional—the first Chinook migrants to arrive each year had slightly lower survival, despite experiencing some of the coolest temperatures. These fish also travelled the slowest, which could account for their higher mortality. Larger cumulative temperature loads prior to entering a reach also lowered survival in the model for both species, with especially strong impacts on sockeye (Fig 2).

Flow effects were more variable, ranging from negative effects on Chinook during the spring freshet in the Columbia and Salmon reaches, to strongly positive effects on sockeye in their final stretch through the Salmon River (note the scale of the x-axis in Fig 3). High flows and high temperatures almost never occur together (note the absence of points in the upper right of Fig 3A–3F), so model projections in that region were unconstrained. Due to sockeye migrating later in the year when flows are lower and more predictably declining, temperatures and flows are more correlated for sockeye than for Chinook. Nonetheless, the flow signal was very strong for sockeye in the Salmon reach within the range of the observed data (Fig 3F).

Non-environmental factors also had significant effects on survival (Fig 2). Snake River sockeye adults that had been transported downstream as juveniles had an observed survival rate through the Columbia reach that was half that of in-river migrants (0.30 vs. 0.59). Fish age was also significant for both species, with ocean-age 1 fish (jacks) in particular demonstrating higher survival through the hydrosystem. This result may be partly explained by selective fishing pressure for older fish, which are generally larger.

#### Arrival-time models

In all model fits for *arrival day* in both species, higher temperatures led to earlier arrival times, while higher flows led to later arrival times. For sockeye, April mean temperature had strong support as the best predictor, with mean arrival day being 1.36 d earlier per degree difference in temperature (S2 Table). For Chinook, April mean temperature was the best predictor of arrival distribution for spring-run, whereas April flow was the best predictor of arrival for summer-run. Arrival was 3.06 d earlier per degree difference in temperature, and 0.02 d later per unit change in flow (km^3^/s, S3 Table). Both sockeye and Chinook models had strong performance, with correlation between predicted and observed quantiles of arrival times at 0.97 and 0.93, respectively.

### Prospective analyses

#### Temperature and flow projections

**Fig 4:**
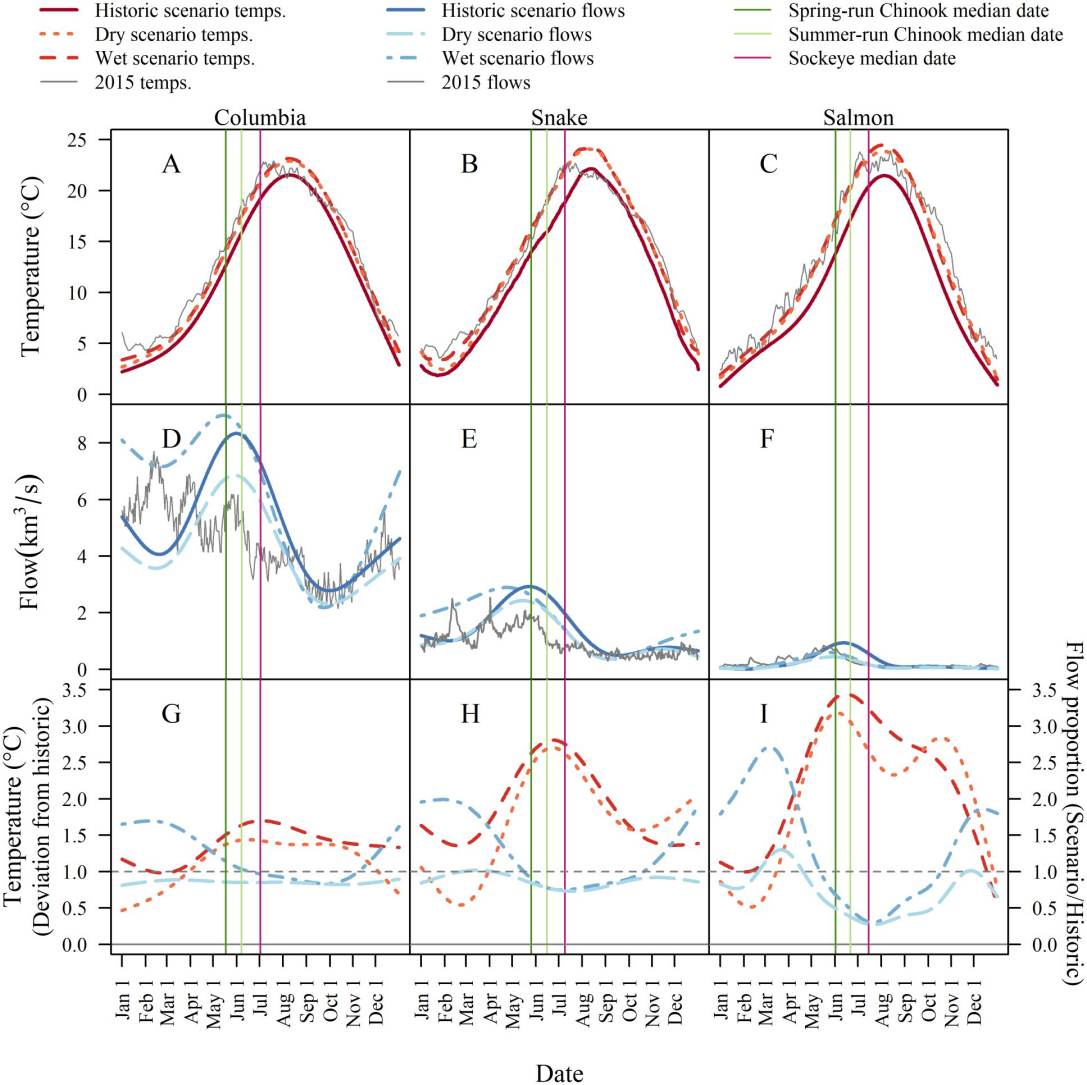
Projected mean daily temperature (A-C) and flow (D-F) in the Columbia, Snake, and Salmon River reaches under three climate scenarios (historical, dry, and wet). The bottom row shows the difference between modeled future and historical daily means. Vertical lines depict current median run dates for spring-run Chinook, summer-run Chinook, and sockeye.

Global climate models consistently project higher air temperatures, which lead to earlier snow melt and thus an earlier spring freshet [46]. The magnitude of spring flows depends on the quantity of winter precipitation and is more variable across climate models than temperature metrics. Our downscaled projections showed the same patterns (Fig 4). Increases in stream temperature were largest in summer, and summer flows decreased in both climate scenarios. However, early-season flows increased in the wet scenario while changing little or decreasing in the dry scenario. Timing of peak flows was earlier in both scenarios.

Compared to other times of the year, changes in temperature during Chinook and sockeye migrations were predicted to be relatively large. The largest predicted temperature changes occurred in June and July (orange and red peaks in Fig 4G–4I), with stressful temperatures arriving earlier in the season (Fig 4A–4C). Mean temperatures increased as fish moved upstream into smaller tributaries (S4 Table), starting with 1.3-1.7 °C at Bonneville (Fig 4G), 2.3-2.8 C at Ice Harbor Dam (Fig 4H), and increasing to 2.6-3.4 °C in the Salmon reach (Fig 4I).

Adult spring Chinook arrive at Bonneville Dam slightly before peak flows, so under the wet scenario, the present run timing would expose these fish to higher mean flows in the Columbia (Fig 4D) but lower flows by the time they reach the Salmon River (Fig 4F). Summer-run Chinook, which arrive at Bonneville about two weeks later, would encounter mean flows similar to those experienced at present. However, summer Chinook would encounter lower flows in upstream reaches in the wet scenario, and lower flows in all reaches in the dry scenario. Sockeye migrate during the descending limb of the spring freshet, and thus would encounter reduced flows in all reaches under both scenarios, with declines in flow of up to 50% during their final leg through the free-flowing Salmon River.

### 2015 represents the “new normal” for temperature and flow

Temperatures during 2015 in the Snake and Salmon reaches were exceedingly hot in spring/early summer but approached average in late July and August before climbing to well above average in fall (Fig 4A–4C). High fall temperatures will affect migrating fall Chinook and steelhead, but not the salmon runs considered in this analysis.

**Fig 5:**
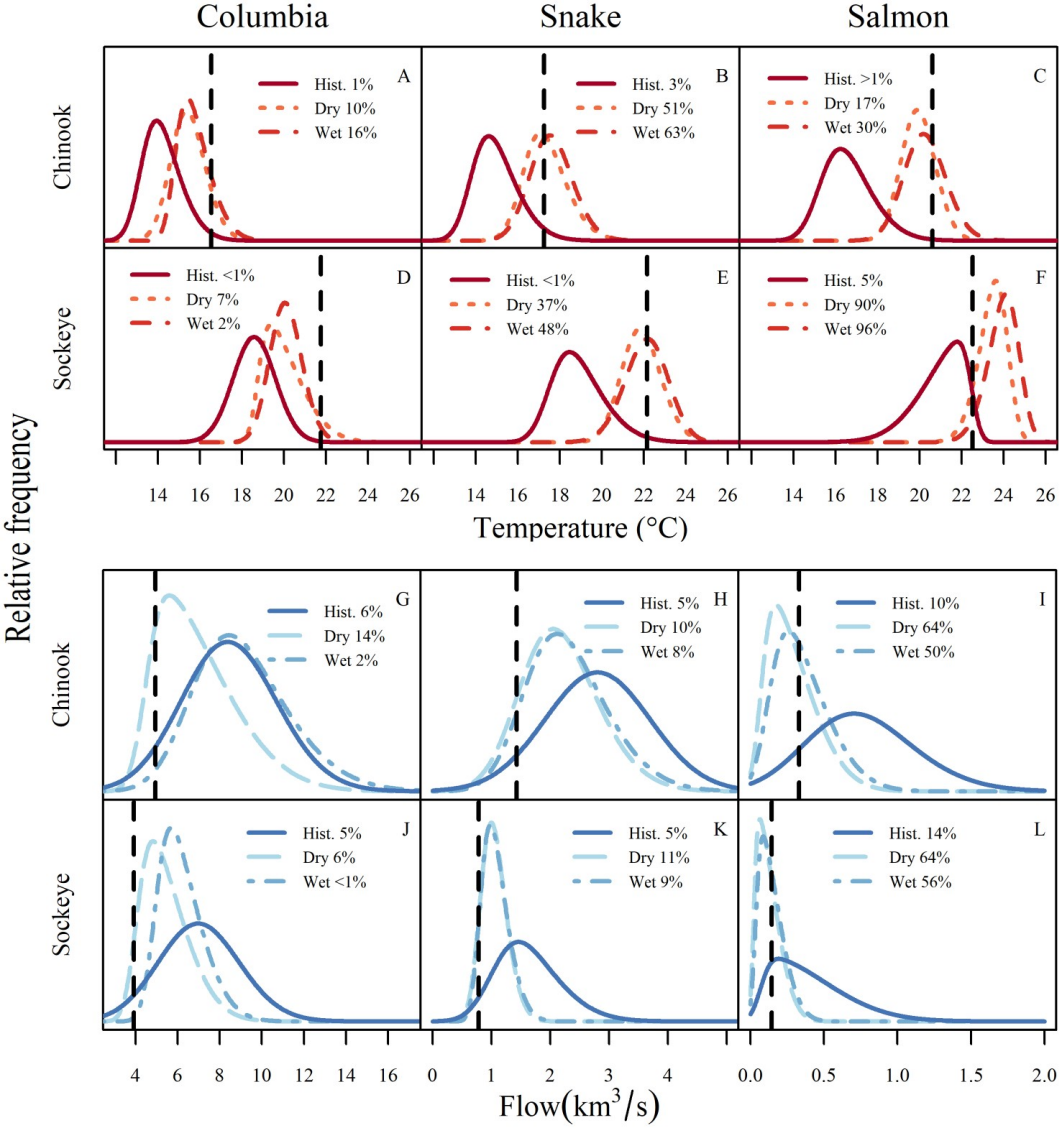
Predicted frequency of mean temperatures (A-F) and flows (G-L) averaged during the Chinook and sockeye runs (5-95^th^ quantiles of observed reach entry dates) for climate scenarios. Means for 2015 are shown as dotted vertical lines. To smooth the distribution of annual temperatures and flows for plotting, we fit a skewed normal distribution for each climate change scenario. The frequency of years as extreme as 2015 is shown in each legend.

To assess how 2015 ranked in relation to interannual variation, we compared mean temperature and flow in 2015 to the means in each year of our simulation scenarios during the present migration period of both species. For migration periods in each year, we used a date range that encompassed the central 95% of each run (Fig 5). Conditions as warm or warmer than those in 2015 were relatively rare in the historical scenario, occurring in fewer than 1 to 5% of years, depending on the species/reach.

However, conditions similar to those of 2015 became more common in future scenarios. In the Columbia reach, these conditions remained uncommon, but became less rare under both scenarios, occurring in 10 and 7% of years in the dry scenario and in 16 and 3% of years in the wet scenario for Chinook and sockeye, respectively. In the Snake River, conditions warmer than 2015 made up a majority of simulation years during the Chinook run (51% dry, 63% wet), and were still very common during the sockeye run (37% dry, 48% wet). Finally, 2015 conditions in the Salmon reach became more common for Chinook (17% dry, 30% wet) and were much cooler than the average future scenario for sockeye (90% dry, 96% wet).

In the historical climate, hydrosystem flows as low as those of 2015 were very infrequent, occurring in less than 6% of simulation years (Fig 5). Such low-flow years occurred in up to 14% of years in the dry scenario in the Columbia and 11% in the Snake. Due to predicted substantial declines in flow in the Salmon River, years at least as dry as 2015 are predicted to become quite common (50-64% across species and future scenarios).

#### Survival projections

**Fig 6:**
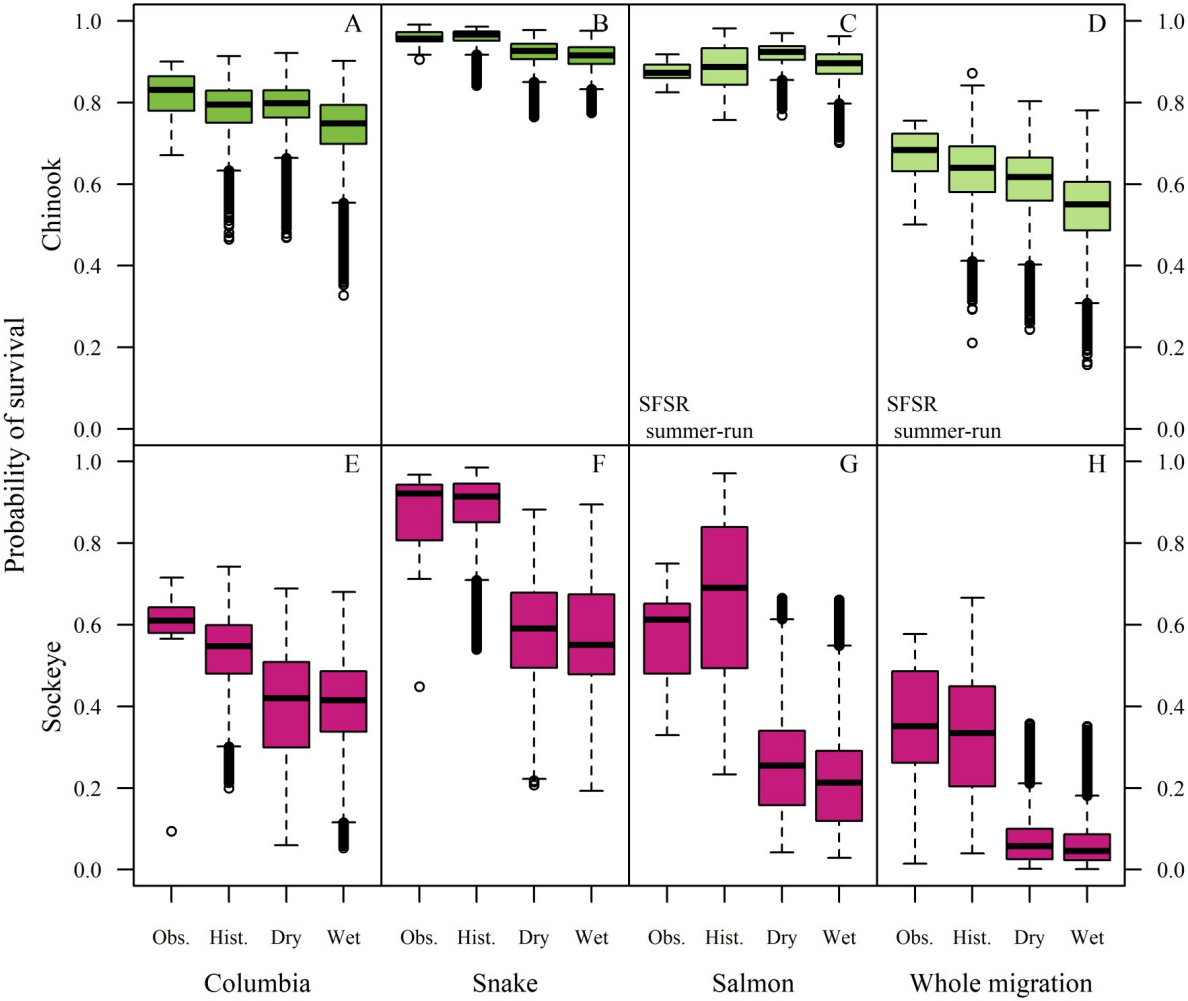
Boxplots of mean annual survival across reaches and climate scenarios. The first column within each panel shows the range of annual survival across years from observed tag data (line = median, box = interquartile range, whiskers = 1.5 × interquartile range). The next three columns show simulated survival across years under the historical, dry, and wet climate scenarios. The final column shows cumulative survival through the entire migration (Bonneville Dam to South Fork Salmon River for Chinook, and to the Sawtooth weir for sockeye).

**Fig 7:**
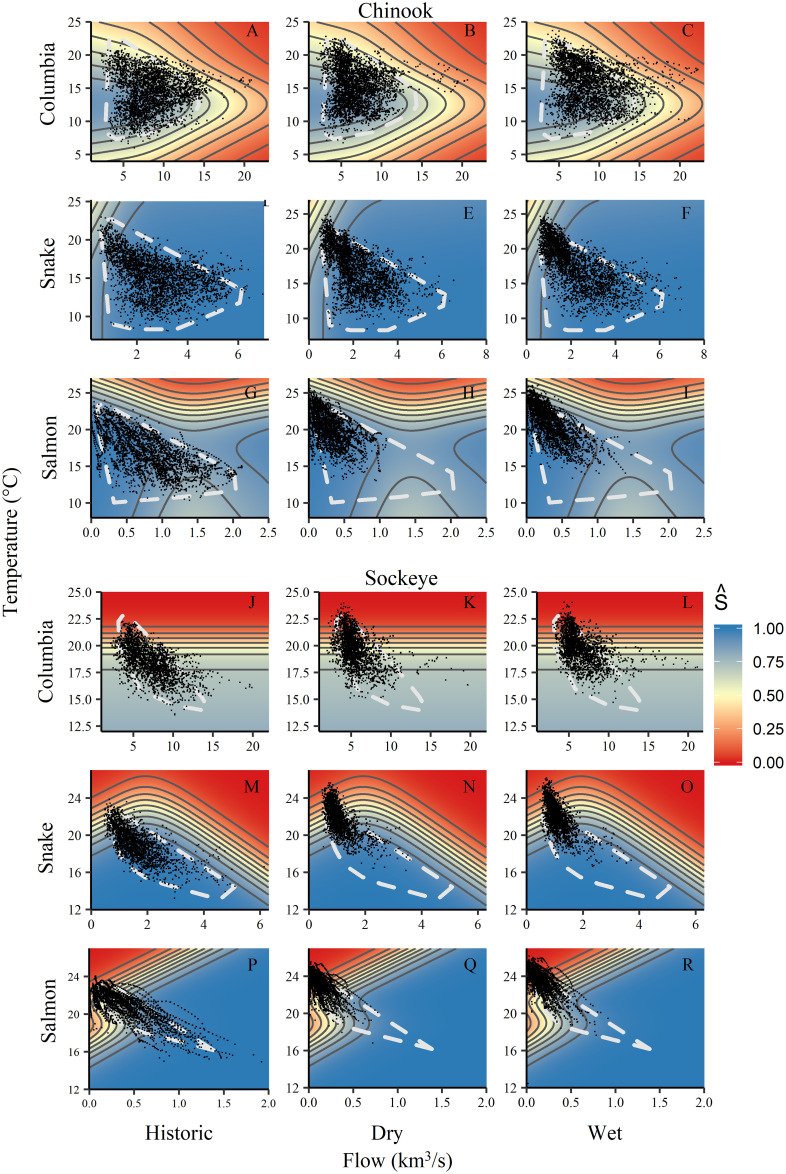
Apparent survival surfaces as a function of temperature and flow from Fig 3 overlaid with simulated fish in the historical, dry, and wet climate scenarios (columns). White polygons show the range of environmental conditions in the observation dataset (from Fig 3) for comparison to the distribution of points in the climate simulations (black). Although some simulated fish experienced temperatures and flows that were outside the range of our observation dataset, the majority of simulated fish experienced conditions within those observed in the retrospective analyses (i.e., most points are inside the polygons). There was also a loss of low temperature and high flow combinations in both climate scenarios (spaces within polygons that lack points), particularly in the Snake and Salmon reaches.

For summer Chinook across the Columbia, Snake, and Salmon reaches, cumulative mean annual survival declined from 63% in historical simulations to 61% in the dry climate, and 54% in the wet climate scenarios (S4 Table, Fig 6). Survival declined less for spring-run than for summer-run Chinook in reaches where they could be compared because of larger temperature impacts on later migrants. Chinook survival declined more in the wet than in the dry scenario because of the detrimental high mainstem flows and slightly higher temperatures projected in the wet scenario (Figs 3 and 7, S2 Table).

In the Salmon reach, summer-run Chinook survived slightly better in both climate scenarios compared with the historical scenario due to positive effects of lower flows on early migrants, which outweighed declines in the survival of late migrants encountering higher temperatures (Fig 7H–7I). Overall, the bulk of the Chinook run remained in suitable conditions, although the later part of the run encountered high temperatures more frequently in both wet and dry climate change scenarios than the historical climate.

Sockeye survival, which is already low, declined much more dramatically, at ~24% in the Columbia, ~35% in the Snake, ~60-65% in the Salmon reach, with cumulative reductions in survival of 78-81% (Table 4). Both climate scenarios demonstrated similar results for sockeye, with cumulative survival below 5% in 41% of individual simulated dry-scenario years and in 55% of wet-scenario years. Effects on sockeye were mostly due to increased temperatures interacting with steep survival gradients, although lower flows in the Salmon River also depressed survival (Fig 7).

**Table 4:** Mean (SD) survival of Chinook and sockeye salmon under simulated climate scenarios vs. observed climate data.

Reach/scenario	Chinook mean survival (SD)			Sockeye	Chinook (%Δ)			Sockeye (% Δ)
	Aggregate	Spring	Summer	mean survival	Aggregate	Spring	Summer	
**Columbia**								
Observed	0.82 (0.06)	0.83 (0.04)	0.80 (0.08)	0.55 (0.21)				
Historical	0.78 (0.06)	0.83 (0.06)	0.75 (0.08)	0.53 (0.09)				
Dry	0.79 (0.06)	0.85 (0.04)	0.75 (0.08)	0.4 (0.13)	0.8%	3.0%	-1.0%	-24.6%
Wet	0.74 (0.08)	0.8 (0.07)	0.69 (0.1)	0.41 (0.11)	-5.9%	-3.6%	-7.8%	-23.4
**Snake**								
Observed	0.96 (0.02)	0.97 (0.02)	0.95 (0.03)	0.83 (0.19)				
Historical	0.96 (0.02)	0.97 (0.01)	0.94 (0.04)	0.88 (0.1)				
Dry	0.92 (0.03)	0.96 (0.02)	0.89 (0.05)	0.59 (0.14)	-4.0%	-0.9%	-6.4%	-34.0%
Wet	0.91 (0.03)	0.96 (0.02)	0.87 (0.05)	0.56 (0.15)	-4.8%	-1.2%	-7.8%	-35.5%
**Salmon**								
Observed			0.87 (0.03)	0.57 (0.16)				
Historical			0.89 (0.05)	0.66 (0.2)				
Dry			0.92 (0.03)	0.27 (0.13)			3.5%	-59.6%
Wet			0.89 (0.04)	0.23 (0.14)			0.2%	-64.8%
**Cumulative**								
Observed			0.65 (0.11)	0.34 (0.17)				
Historical			0.63 (0.08)	0.33 (0.15)				
Dry			0.61 (0.08)	0.07 (0.06)			-4.0%	-77.8%
Wet			0.54 (0.09)	0.06 (0.06)			-14.6%	-80.9%

Aggregate applies the full mixture model to Chinook salmon, whereas spring and summer components are tracked separately in subsequent columns. No spring Chinook populations had sufficient upstream detection rates to develop a model of survival in the Salmon reach. South Fork Salmon River populations were modeled to represent summer-run Chinook in the Salmon reach. The Δ columns show percent change in survival from historical to wet and dry climate change scenarios.

### Sensitivity analyses

**Fig 8:**
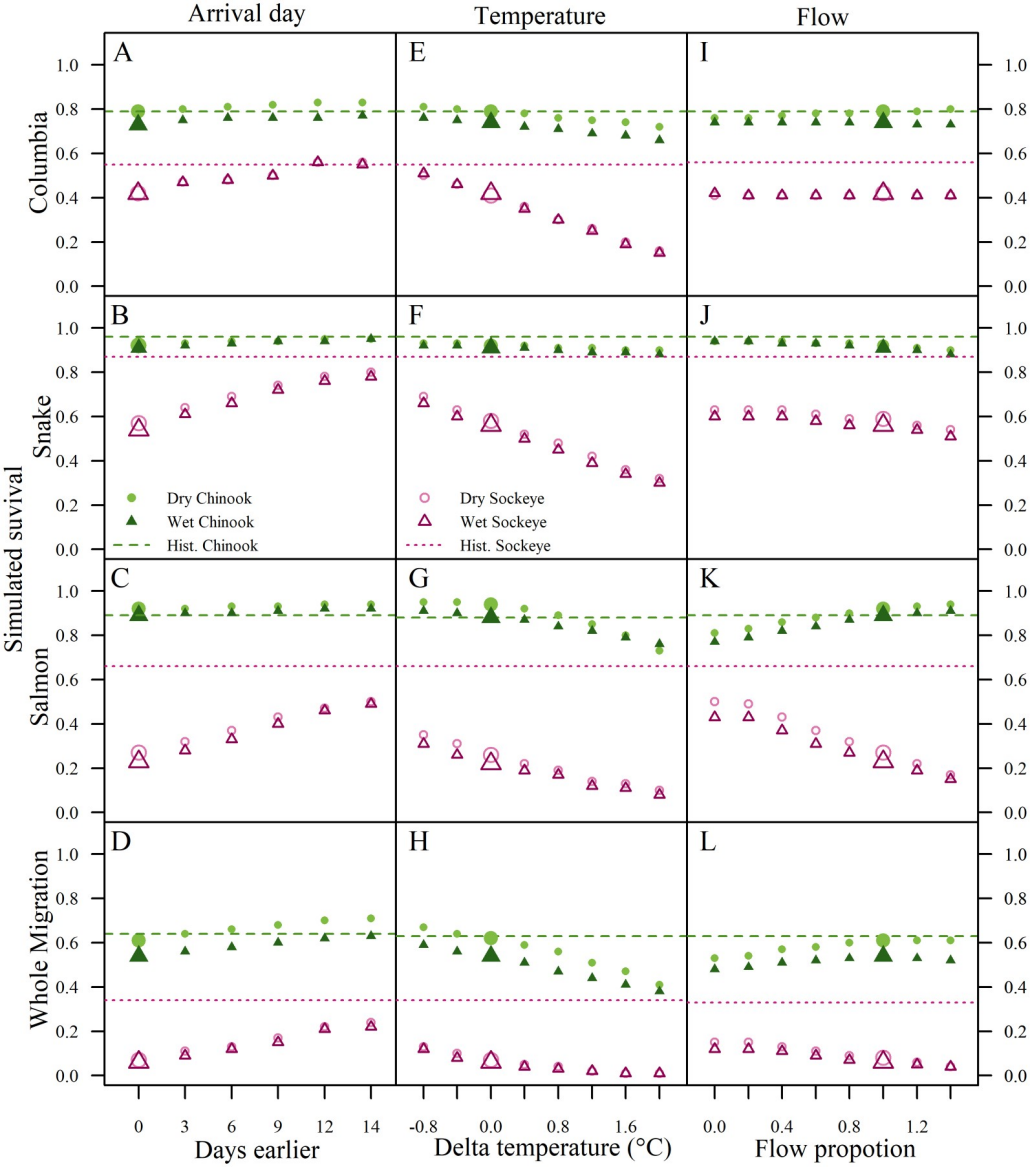
Sensitivity analysis of reach survival estimates for Chinook and sockeye. We explored the wet and dry scenarios with additional alterations to migrating timing (A-D), temperature change (E-H), and flow (I-L). The larger symbols represent the base climate scenario survival predictions with no changes to parameters (Table 4). Horizontal lines show the mean survival rates in the historical climate scenario for each species.

Sensitivity analyses suggested that a large additional shift (>2 weeks) in migration timing would be needed over the 1.5 d expected response for sockeye to compensate for either the wet or dry climate change scenario (Fig 8D). Chinook survival was less sensitive to arrival timing than sockeye, but still would benefit slightly from a shift to earlier migration beyond the ~3 d expected based on the arrival timing model (Fig 8D). Results in all reaches were sensitive to temperature change; larger temperature increases led to lower survival estimates for both species in all reaches, but especially for sockeye in the Columbia and Snake reaches (Fig 8E and 8F). For flow, note that in our analyses, we explored proportional change to the delta value imposed by the scenario for a particular day, but did not change the direction of the change. In other words, proportions below 1 dampened the blue curves in while those above 1 had the opposite effect (e.g., flattening vs. amplifying the curves in Fig 4G–4I). Survival results showed that most reaches were less sensitive to changes in the flow scenario than changes in temperature, with mixed responses in Chinook (Fig 8I–8L). For sockeye, however, which encounter lower flows throughout the migration in both the wet and dry scenarios, reducing the magnitude of predicted declines in flows in the Salmon River was particularly beneficial for survival (Fig 8K).

## Discussion

For migrating adult salmon in the Columbia and Snake River Basin, model-projected changes in flow and temperature for the 2040s may reduce survival somewhat for Chinook, but will likely have severe impacts on anadromous sockeye. In most years, rising temperatures rather than altered flows had the most damaging effects on sockeye, with the majority of adults expected to confront temperatures exceeding 19°C at Bonneville, 21°C at Ice Harbor Dam, and 23°C in the lower Salmon River. In some cases, major declines in summer flow exacerbated the impacts of temperature change, particularly in the free-flowing section of the migration route upstream of the hydrosystem. Such high temperatures have already been associated with high mortality in these endangered fish [32, 39], and were likely the culprit of the major run collapse in 2015.

### Anthropogenic effects increased sockeye sensitivity to climate change

Higher sensitivity of sockeye than of spring/summer Chinook in our analyses reflected both intrinsic life history characteristics and responses to anthropogenic factors. Adult sockeye have evolved to migrate upstream during mid-summer, which inherently exposes them to higher temperatures and lower flows than spring Chinook experience during their migration. Because of this, sockeye are more susceptible to compounded impacts of warmer water and lower summer flows from climate change [19].

Snake River sockeye also demonstrated much lower adult survival among fish transported downstream as juveniles. For these fish, observed survival through the Columbia reach was half that of in-river migrants (0.30 vs. 0.59). The difference in survival of transported fish vs. in-river migrants was much larger for sockeye than that modeled for adult Chinook, which had only 3% lower survival for transported fish in our study and 10% observed by Keefer, Caudill [49]. Effects of juvenile transportation are generally thought to result from impaired homing ability [50]. As adults, transported fall Chinook and steelhead have a stronger tendency to stray into non-natal tributaries than their in-river migrant cohorts [51, 52]. Some fish also fall back downstream after passing a dam and then re-ascend. Fallback has not been a significant predictor of survival for Chinook in previous analysis [53], but occurred at much higher rates in sockeye and has been a significant predictor of sockeye survival [28]. Fallback also slows travel and can increase cumulative temperature load.

For both species, larger cumulative temperature loads prior to entering a reach lowered survival in the model, with especially strong impacts on sockeye (Fig 2). Cumulative temperatures reflect the combination of migration rates and temperature, so this result suggests that any migration delay exacerbates mortality, especially when temperatures are high. High temperatures can delay migration directly [54–56], although the populations considered here have not yet shown this behavior. Many other influences can slow migration, including high spill at dams, encounters with predators and fisheries, difficulty finding or entering fishways, and turbulence or eddies in tailraces [57].

Higher sensitivity to anthropogenic factors for Snake River sockeye vs. Chinook could reflect cumulative effects in the former from some two decades of captive rearing. This history includes a precipitous decline followed by complete dependence of the population on restoration hatchery production. The magnitude of this hatchery effect is not quantifiable because of the absence of wild fish for comparison. Nonetheless, for adult migrants returning to the upper Columbia River, comparisons of fallback rate and survival between wild and hatchery sockeye [28] have shown differences similar to those observed in comparisons of wild vs. hatchery Chinook [53]. Many other studies have documented lower survival in hatchery compared with wild fish [58, 59]. Thus, our observations support the existing literature showing a compounding influence of pre-existing conditions on vulnerability to climate change.

Our survival estimates reflect projected run timing, the combined effects of temperature and flow in survival models, and specific projections for climate change. There is uncertainty in each of these components. However, our sensitivity analyses and comparisons with other studies support our conclusions. For example, we assumed only plastic changes in our arrival timing model, although evolutionary change has been observed [39, 60] and projected in other sockeye salmon populations [61]. At present, nearly all Snake River sockeye are produced from captive broodstock, which is not subject to natural selection on adult run timing. Artificial selection on migration timing could be imposed [62], but carries the risk of maladaptive spawn timing, along with additional unintended consequences [59]. Our sensitivity analyses showed that Snake River sockeye would still encounter very high temperatures in the Salmon River, even if they arrived two weeks earlier than predicted. Thus, selection for run timing would not adequately compensate for impacts of climate change across the entire migration (Fig 8).

Our climate projections stemmed from a limited number of GCM scenarios, but were similar to those projected by other researchers using a wide variety of methods [63–65]. Over timescales similar to ours, projected increases in water temperature for the mainstem Columbia and Snake Rivers have generally ranged 1-3°C, with reductions in flow ranging 0-30%, depending on the location and season of interest.

Our cumulative temperature estimates for sockeye overlapped with those of Isaak, Luce [64], but our projected survival rates were slightly higher because our model accounted for expected shifts in migration speed and in arrival day at Bonneville Dam. Our sensitivity analyses showed that more warming, which is likely if carbon emissions are not curtailed, would be harmful to both species (Fig 8). Our cumulative migration results were only moderately sensitive to the flow scenario, where greater uncertainty lies in climate models.

Analyses in this study focused on a single life stage, but ultimately full life cycle analyses are necessary to account for carryover effects between life stages and for cumulative effects at all life stages from both climate change and management actions. Carryover effects occur when conditions in a previous life stage affect performance in a subsequent life stage [66]. We considered carryover effects of juvenile transportation on the adult migration, but not total effects across the lifetime. In Chinook, despite negative effects in the adult life stage, the net effects of juvenile transportation on lifetime survival are still positive under certain circumstances [67–69]. However, adult returns of sockeye have been too low to rigorously assess these lifetime effects [70].

An additional carryover effect that we did not explicitly account for was the physiological condition of adult fish at freshwater entry. Early ocean growth rates vary widely with ocean conditions, and hatchery Chinook may be less able to compensate for the annual change in prey fields than wild Chinook [71]. However, the extent to which ocean conditions might affect adult migratory stages, especially in sockeye, is still unclear. The random effect we included for year might partially reflect ocean conditions, but further work is needed to explore potential carryover effects from favorable vs. unfavorable ocean ecosystem conditions.

Carryover effects should also be considered from migration to subsequent stages. Chinook salmon in particular, could have post-migration difficulty finding deep, cool pools in which to hold prior to spawning [72–74]. Spring Chinook were expected to advance their migration timing and migrate faster in response to higher temperatures, increasing the total holding period. Bioenergetic costs of holding are extremely sensitive to temperature [75] and the duration of holding. Total energetic costs also depend on spawn date, which could shift later due to higher temperatures in early fall. Consequently, lower flows and higher temperatures may stress adult Chinook that have completed their migration, increasing pre-spawn mortality. For Snake River sockeye, we expect less change in pre-spawn mortality because they return to a deep, cool lake in which hold over summer. Therefore, major biological costs for sockeye will likely accrue during the migration stage.

### System-specific recommendations

For Snake River sockeye salmon, recovery goals entail restoring the ESU to self-sustaining, anadromous, wild populations [76]. Increased mortality in the migratory stage threatens this goal. The greatest declines in sockeye survival were projected for the Salmon reach, where major declines in snowpack led to lower flows and higher temperatures. Specific actions have been identified in the recovery plan to address impaired water quality due to land use practices and irrigation diversions (Table 7–1 in [76]). In some areas of critical habitat for these fish, contaminants that impair fitness are common, such as insecticides and herbicides from agricultural runoff and heavy metals from mine waste [70]. For Snake River Chinook, habitat actions that augment summer holding habitat might also be needed to mitigate the risk of pre-spawn mortality (e.g., increasing deep pool holding habitat or riparian shading to mitigate stream temperatures).

Given that climate change presents a dire threat to anadromous sockeye from this ESU, addressing these problems is urgent. Short-term solutions include collecting adult sockeye at Lower Granite Dam for transport to the Sawtooth Valley. Although pilot experiments have been successful, substantial advances would be required to transport numbers sufficient to maintain the natural population. Mortality risks and difficult logistics appear daunting, but methodologies and risk-assessment tools have been developed to enhance potential benefits of trap and haul (Colvin et al 2018; Lusardi and Moyle 2017). However, the vast majority of Snake River sockeye died before reaching Lower Granite Dam in 2015. Thus, if 2015 represents future conditions in the lower Columbia, transportation would have to be initiated further downstream to be beneficial.

In the Columbia Basin, nearly all hydrosystem planning has focused on flow and spill rather than temperature [41, 77], despite the enormous sensitivity of salmonids to temperature. A coordinated flow management plan could reduce some impacts of climate change, but supporting analyses that include temperature have not been completed. Some of the most serious temperature effects are expected to occur in tributaries upstream of major hydrosystems, where local recovery actions are needed. Temperature-induced changes in survival for adult Chinook were on the same order of magnitude as harvest, suggesting that there are management levers that could compensate for climate change in this life stage. However, climate change will likely become an increasing challenge over time for both species.

## Conclusions

Many of the species already most affected by anthropogenic factors may be the most vulnerable to climate change. Adaptive capacity is already limited in these species, and extra effort is urgently needed to compensate for this handicap. Full assessment of climate impacts requires consideration of the full life cycle, but stage-specific effects will likely vary by life history type and spatial distribution. Comprehensive analyses are not possible everywhere, but downscaled projections of future climate conditions are increasingly available for ecological models. Crucial areas of uncertainty for specific management decisions can be effectively clarified by using thorough sensitivity analyses and appropriate propagation of parameter uncertainty through a simulation approach.

Extreme temperatures and drought in early 2015 constituted a stress test of migration conditions in the Columbia River Basin that may be a harbinger of typical conditions in the near future [78]. We can expect many more years as warm as 2015, but in the future, abnormally high temperatures will likely continue throughout summer rather than abating as they did in 2015. Furthermore, projections display much greater spatial homogeneity in future years than was observed in 2015 [79], putting many more localities at high risk. As record-breaking climatic conditions occur more frequently, careful examination of the complex environmental and anthropogenic changes that threaten existing biodiversity is a growing conservation imperative.

## Supporting information

S1 Table Apparent survival by year for each species and run of Chinook and sockeye salmon. (DOCX)

S2 Table Arrival timing model selection table for sockeye salmon showing the environmental covariate (temp. = temperature), model parameters (B0 = intercept, B1 = slope, SD = standard deviation), and delta AICc values. We selected the model with the lowest AICc (in bold).
(DOCX)

S3 Table Arrival timing model selection table for Chinook salmon showing the environmental covariate and model parameters for the spring-run (spr) and summer-run (su) components respectively (Eq 3), and the delta AICc values for each model. We selected the model with the lowest AICc (in bold).
(DOCX)

S4 Table Mean conditions experienced by observed fish and used in climate simulations. Variables shown include mean temperatures (*T*) and flows (*F*), arrival day of year (*D*), proportion transported (*J*), proportion hatchery (*H*), average ocean age (*A*), and catch (cube root transformed). Note, Salmon River variables are for observed and simulated summer-run Chinook from the SFSR.
(DOCX)

S1 File (ZIP)
