# Supplementary material for: Snake River sockeye and Chinook salmon in a changing climate: Implications for upstream migration survival during recent extreme and future climates
Source: PLoS One. 2020 Sep 30;15(9):e0238886. doi: 10.1371/journal.pone.0238886 (PMC7526937; doi:10.1371/journal.pone.0238886)
Supplement: S1 Table — (DOCX) [file pone.0238886.s001.docx]

### S1 Table. Apparent survival by year for each species and run of Chinook and sockeye salmon.
