# Supplementary material for: Snake River sockeye and Chinook salmon in a changing climate: Implications for upstream migration survival during recent extreme and future climates
Source: PLoS One. 2020 Sep 30;15(9):e0238886. doi: 10.1371/journal.pone.0238886 (PMC7526937; doi:10.1371/journal.pone.0238886)
Supplement: S2 Table — We selected the model with the lowest AICc (in bold). (DOCX) [file pone.0238886.s002.docx]

### S2 Table: Arrival timing model selection table for sockeye salmon showing the environmental covariate (temp. = temperature), model parameters (B0=intercept, B1=slope, SD=standard deviation), and delta AICc values. We selected the model with the lowest AICc (in bold).

| **Variable** | **B0** | **B1** | **SD** | **ΔAICc** |
| --- | --- | --- | --- | --- |
| **April temp.** | 196 | -1.360 | 7.4 | 0.0 |
| April-May temp. | 195 | -1.008 | 7.4 | 14.5 |
| June flow | 181 | 0.008 | 7.5 | 20.3 |
| May temp. | 193 | -0.754 | 7.5 | 25.2 |
| May-June flow | 181 | 0.009 | 7.5 | 28.1 |
| May -June temp | 191 | -0.491 | 7.5 | 34.5 |
| June temp. | 189 | -0.349 | 7.5 | 39.8 |
| May flow | 181 | 0.009 | 7.5 | 40.5 |
| April-May flow | 181 | 0.007 | 7.5 | 49.8 |
| April flow | 182 | 0.003 | 7.5 | 57.8 |
