# Supplementary material for: Snake River sockeye and Chinook salmon in a changing climate: Implications for upstream migration survival during recent extreme and future climates
Source: PLoS One. 2020 Sep 30;15(9):e0238886. doi: 10.1371/journal.pone.0238886 (PMC7526937; doi:10.1371/journal.pone.0238886)
Supplement: S3 Table — We selected the model with the lowest AICc (in bold). (DOCX) [file pone.0238886.s003.docx]

### S3 Table: Arrival timing model selection table for Chinook salmon showing the environmental covariate and model parameters for the spring-run (spr) and summer-run (su) components respectively (Eq. 3), and the delta AICc values for each model. We selected the model with the lowest AICc (in bold).

| Spring variable | Summer variable | p | BO.spr | B1.spr | B0 su | B1.su | SD.spr | SD.su | ΔAICc |
| --- | --- | --- | --- | --- | --- | --- | --- | --- | --- |
| **April temp.** | **April flow** | **0.37** | **163** | **-3.059** | **159** | **0.019** | **13.9** | **11.7** | **0.0** |
| April temp. | April-May flow | 0.34 | 163 | -3.049 | 159 | 0.016 | 13.8 | 11.8 | 9.9 |
| April temp. | May flow | 0.33 | 161 | -2.899 | 160 | 0.011 | 13.6 | 11.9 | 23.3 |
| April temp. | April temp. | 0.30 | 162 | -2.972 | 172 | -1.008 | 13.5 | 11.9 | 25.5 |
| May flow | April flow | 0.30 | 125 | 0.032 | 159 | 0.019 | 13.3 | 11.8 | 26.5 |
| May flow | April-May flow | 0.28 | 125 | 0.032 | 159 | 0.017 | 13.2 | 11.9 | 36.3 |
| April temp. | May-June flow | 0.30 | 157 | -2.463 | 161 | 0.006 | 13.5 | 11.9 | 36.8 |
| April temp. | June flow | 0.33 | 162 | -2.932 | 161 | 0.006 | 13.7 | 11.9 | 37.0 |
| April-May flow | April flow | 0.35 | 128 | 0.026 | 160 | 0.018 | 13.7 | 11.7 | 38.5 |
| April temp. | April-May temp. | 0.33 | 162 | -3.008 | 171 | -0.674 | 13.7 | 11.9 | 38.9 |
