# Supplementary material for: Snake River sockeye and Chinook salmon in a changing climate: Implications for upstream migration survival during recent extreme and future climates
Source: PLoS One. 2020 Sep 30;15(9):e0238886. doi: 10.1371/journal.pone.0238886 (PMC7526937; doi:10.1371/journal.pone.0238886)
Supplement: S4 Table — Variables shown include mean temperatures (T) and flows (F), arrival day of year (D), proportion transported (J), proportion hatchery (H), average ocean age (A), and catch (cube root transformed). Note, Salmon River variables are for observed and simulated summer-run Chinook from the SFSR. (DOCX) [file pone.0238886.s004.docx]

### S4 Table: Mean conditions experienced by observed fish and used in climate simulations. Variables shown include mean temperatures (*T*) and flows (*F*), arrival day of year (*D*), proportion transported (*J*), proportion hatchery (*H*), average ocean age (*A*), and catch (cube root transformed). Note, Salmon River variables are for observed and simulated summer-run Chinook from the SFSR.

|  | Observed | Historical | | Dry | Wet |
| --- | --- | --- | --- | --- | --- |
|  | *Chinook* | | | | |
| *T Columbia* | 14.31 (2.46) | 14.49 (2.65) | 15.46 (2.81) | | 15.84 (2.87) |
| *T Snake* | 14.33 (2.43) | 15.41 (2.40) | 17.52 (2.93) | | 17.93 (2.97) |
| *T Salmon* | 16.80 (3.14) | 16.83 (2.70) | 20.12 (2.91) | | 20.68 (3.08) |
| *F Columbia* | 8.16 (2.67) | 8.51 (2.92) | 6.82 (2.47) | | 8.85 (2.98) |
| *F Snake* | 2.74 (1.23) | 2.78 (1.19) | 2.1 (0.98) | | 2.16 (1.16) |
| *F Salmon* | 0.84 (0.49) | 0.81 (0.44) | 0.31 (0.39) | | 0.35 (0.39) |
| *D Columbia* | 150.70 (19.08) | 150.59 (18.53) | 148.24 (20.14) | | 149.33 (20.69) |
| *D Snake* | 159.10 (19.29) | 160.37 (18.26) | 157.52 (19.75) | | 158.74 (20.19) |
| *D Salmon* | 171.23 (15.88) | 175.50 (18.02) | 174.03 (19.42) | | 162.54 (19.85) |
| *J* | 0.33 | 0.35 | 0.35 | | 0.35 |
| *H* | 0.74 | 0.72 | 0.72 | | 0.72 |
| *A* | 1.82 | 1.88 | 1.87 | | 1.88 |
| *C* | 1.06 | 1.02 | 1.01 | | 1.00 |
|  |  |  |  | |  |
|  | *Sockeye* | | | | |
| *T Columbia* | 18.69 (2.33) | 18.48 (1.37) | 19.72 (1.4) | | 19.75 (1.27) |
| *T Snake* | 18.37 (1.77) | 19.03 (1.59) | 21.70 (1.38) | | 21.9 (1.47) |
| *T Salmon* | 21.03 (1.28) | 20.77 (1.59) | 23.06 (1.13) | | 23.56 (1.18) |
| *F Columbia* | 7.20 (2.98) | 7.10 (2.43) | 5.61 (1.85) | | 6.52 (2.00) |
| *F Snake* | 1.85 (0.92) | 1.59 (0.65) | 1.07 (0.37) | | 1.09 (0.35) |
| *F Salmon* | 0.34 (0.17) | 0.43 (0.29) | 0.15 (0.11) | | 0.17 (0.12) |
| *D Columbia* | 183.23 (7.52) | 182.87 (7.51) | 180.82 (7.50) | | 180.81 (7.48) |
| *D Snake* | 190.25 (7.90) | 191.43 (7.86) | 188.94 (7.83) | | 188.96 (7.71) |
| *D Salmon* | 195.46 (7.83) | 195.05 (7.85) | 192.23 (7.82) | | 192.22 (7.70) |
| *J* | 0.38 | 0.31 | 0.31 | | 0.31 |
| *H* | 0.99 | 0.99 | 0.99 | | 0.99 |
| *A* | 1.95 | 2.01 | 2.01 | | 2.01 |
| *C* | 1.62 | 1.57 | 1.56 | | 1.56 |
